# Belladonna Versus Opium, and Opium Versus Belladonna

**Published:** 1873-07

**Authors:** 


Belladonna versus Opium, and Opium versus Belladonna.—Reports are frequently found in our exchanges of the antidotal effect of belladonna for opium poisoning, and, from the symptoms detailed, we feel very sure that in more than one instance the antidote proved to be the greater poison. A little caution seems to be necessary, lest the patient be killed by the remedy.

Dr. H. S. Burritt, of Bridgeport, Conn., gives, in the *Reporter*, the details of a cure in which an ounce of laudanum had been swallowed by a man 40 years old, an hour before seeing him. Patient unable to stand, stupid, pupils contracted, etc. No vomiting, from large doses of antimony and ipecac, occurring in half an hour, gave a teaspoonful fluid extract of belladonna, and repeated in ten minutes, after which he (fortunately) had free vomiting. Two hours after, gave another teaspoonful of the fluid extract of belladonna, (Squibb's,) and repeated in an hour and a half. Two and a half hours after the last dose "the scene was entirely changed." "Pupils large, cold, pulse feeble, hiccough, respirations three to the minute, and gasping." Two fluid ounces liquid ammonia and six of whisky were given by injection. One hour after, pulse was full, heat improved. In a few hours became restless and delirious, which was controlled by morphia. The patient recovered. In this case the poison of opinm and of belladenna were relieved, one by the other. The symptoms after the "scene was changed" were more alarming than before. "All is well," however, "that ends well."

Instant Arrest of Epistaxis.—Dr. Marin, of Geneva, states in the *Journal de Med. et de Chirurg. Practique*, May, 1872, that, as bleeding in epistaxis generally flows from only one nostril, and most frequently from the anterior third of one of the nasal fossæ, he was led to believe that, by compressing the corresponding facial artery on the superior maxillary bone, near the ala of the nose, the afflux of blood would be diminished, and the hemorrhages at once be arrested. He has tried the plan in very many serious hemorrhages from the nose, and the expedient has proved perfectly and promptly successful.—*L' Union Medicale.*

Case of Eclampsia Cured by Subcutaneous Injection of Atropia and Morphia.—Puerperal eclampsia; no albumen in the urine; fits violent and rapid; injection with a solution of atropia and acetate of morphia; seven hours' sleep; no return of violent fits; only a few convulsive movements on the following day, with feeling of dryness in throat. Recorded by Dr. Divet in *Gazette de Joulin*, No. 2.—*London Lancet.*

Baron von Leibig, the world-renowned chemist, died at Munich, April 19th, 1873. He was seventy years of age.

